# Development and Validation of the RCOS Prognostic Index: A Bedside Multivariable Logistic Regression Model to Predict Hypoxaemia or Death in Patients with SARS-CoV-2 Infection

**DOI:** 10.1155/2022/2360478

**Published:** 2022-04-20

**Authors:** Gerardo Alvarez-Uria, Sumanth Gandra, Venkata R. Gurram, Raghu P. Reddy, Manoranjan Midde, Praveen Kumar, Ketty E Arce

**Affiliations:** ^1^Department of Infectious Diseases, Rural Development Trust Hospital, Bathalapalli, AP, India; ^2^Division of Infectious Diseases, Department of Internal Medicine, Washington University School of Medicine, St. Louis, Missouri, USA; ^3^Department of General Medicine, Rural Development Trust Hospital, Bathalapalli, AP, India; ^4^Department of Microbiology, Rural Development Trust Hospital, Bathalapalli, AP, India; ^5^Department of Orthopaedics, Rural Development Trust Hospital, Bathalapalli, AP, India; ^6^Department of Emergency Medicine, Rural Development Trust Hospital, Bathalapalli, AP, India

## Abstract

**Introduction:**

Previous COVID-19 prognostic models have been developed in hospital settings and are not applicable to COVID-19 cases in the general population. There is an urgent need for prognostic scores aimed to identify patients at high risk of complications at the time of COVID-19 diagnosis.

**Methods:**

The RDT COVID-19 Observational Study (RCOS) collected clinical data from patients with COVID-19 admitted regardless of the severity of their symptoms in a general hospital in India. We aimed to develop and validate a simple bedside prognostic score to predict the risk of hypoxaemia or death.

**Results:**

4035 patients were included in the development cohort and 2046 in the validation cohort. The primary outcome occurred in 961 (23.8%) and 548 (26.8%) patients in the development and validation cohorts, respectively. The final model included 12 variables: age, systolic blood pressure, heart rate, respiratory rate, aspartate transaminase, lactate dehydrogenase, urea, C-reactive protein, sodium, lymphocyte count, neutrophil count, and neutrophil/lymphocyte ratio. In the validation cohort, the area under the receiver operating characteristic curve (AUROCC) was 0.907 (95% CI, 0.892–0.922), and the Brier Score was 0.098. The decision curve analysis showed good clinical utility in hypothetical scenarios where the admission of patients was decided according to the prognostic index. When the prognostic index was used to predict mortality in the validation cohort, the AUROCC was 0.947 (95% CI, 0.925–0.97) and the Brier score was 0.0188.

**Conclusions:**

The RCOS prognostic index could help improve the decision making in the current COVID-19 pandemic, especially in resource-limited settings with poor healthcare infrastructure such as India. However, implementation in other settings is needed to cross-validate and verify our findings.

## 1. Introduction

Infection with severe acute respiratory syndrome coronavirus 2 (SARS-CoV-2) has high morbidity and mortality [1]. Since late 2019, the rapid spread of SARS-CoV-2 has put enormous pressure on national health systems worldwide.

The clinical spectrum of coronavirus disease 2019 (COVID-19) produced by SARS-CoV-2 is wide. In a large study from China including 72314 cases, 81% had mild disease, 19% had severe disease with deterioration of the respiratory function, and 2.3% died [2]. In many medical domains, prognostic multivariable prediction models have been developed with the aim to help healthcare professionals in their decision making [3]. To date, more than 100 COVID-19 prognostic models have been reported [4]. However, the vast majority of them have been developed in overwhelmed hospital settings from developed countries where the mortality and the proportion of patients with severe disease were high, and they might not be applicable to COVID-19 cases in the general population.

The objective of this study was to develop and validate a pragmatic prognostic score to predict the risk of mortality or hypoxaemia in patients with COVID-19 who were admitted to a hospital regardless of the severity of their symptoms. We hypothesized that the population of the study is similar to the general population of COVID-19 cases, and that the prognostic score could be applied in nonhospital settings, where the majority of cases are mild and the proportion of severe cases is relatively small.

## 2. Methods

### 2.1. Source of Data and Participants

The Rural Development Trust (RDT) COVID-19 observational study (RCOS) is a retrospective observational study of patients diagnosed with COVID-19 and admitted from April 17, 2020, to November 19, 2020, to the RDT General Hospital in Bathalapalli, Anantapur District, Andhra Pradesh, India. During this time, the hospital was designated as a COVID-19 centre and was utilized exclusively to treat patients who had a positive SARS-CoV-2 reverse transcriptase polymerase chain reaction (RT-PCR) or antigen test. During this time, to reduce the risk of transmission in the community, patients with mild symptoms were also admitted for isolation. As per Government rules, even patients with mild symptoms could not be discharged at least until 10 days passed from the symptom onset or the first positive SARS-CoV-2 test.

For the study, we used routinely collected data (demographics, laboratory investigations, and vitals) entered into the hospital information system (HIS). Comorbidities of patients were not entered into the HIS and, therefore, could not be used in the prognostic models. The study was performed according to the principles of the Declaration of Helsinki. The associated Ethics Committee of RDT Bathalapalli Hospital approved the study and waived the need for informed consent. The methodology of the study followed the guidelines for transparent reporting of a multivariable prediction model for individual prediction or diagnosis (TRIPOD) [5]. For the sample size, we took a practical approach by using all available data to maximize the power of the statistical analysis [6].

### 2.2. Outcome and Independent Predictors

We aimed to develop a prognostic model with variables collected at the time of hospital admission that could be utilized to identify COVID-19 patients who were at higher risk of complications. We decided to use a composite endpoint including in-hospital mortality or hypoxaemia as the primary outcome of the study. Hypoxaemia was defined as having oxygen saturation below 93% or the need for oxygen support to maintain saturation above 93% [2, 7]. We selected *a priori* set of potential predictors according to the availability of data in the HIS and whether the variables had shown to influence the outcome of COVID-19 in previous studies [8–13].

### 2.3. Model Development and Validation

The dataset was split in two. Development of the model was performed with data from patients admitted from April 17 to August 31, 2020 (development cohort), while model validation was performed with patients admitted from September 1 to November 19, 2020 (validation cohort).

Assuming missing at random, missing values were imputed using chained equations in 10 datasets, each with 10 iterations [14, 15]. The outcome was included as a predictor in the imputation of the development cohort but not in the validation cohort.

Because our primary objective was to develop a pragmatic bedside risk score that did not demand complex calculations, we decided to categorize continuous variables [12]. Model development was performed in four stages. In the first stage, we made an initial selection of predictors based on the goodness of fit between the outcome and predictors using generalized additive models (GAMs). Categorical predictors were entered as linear components in the models, and continuous predictors were smoothed using penalized thin-plate splines [16]. In the second stage, we selected optimal cutoff values to categorize continuous variables based on visual inspection of the GAM models [17]. In the third stage, to reduce the risk of model overfitting, we used least absolute shrinkage and selection operator (LASSO) regression with theory-driven penalization to select predictors and their cutoff values to be included in the final model [18]. In the fourth stage, we used the coefficients from the logistic regression models to construct the prognostic index.

One common problem when comparing laboratory data is that laboratory values are highly dependent on the methodology used, and data normalization is needed. Usually, clinical laboratories have normal ranges that enclose 95% of values in a healthy population. In settings where the laboratory normal range differs substantially from our values, we recommend using the lower or upper normal limits of their laboratory as the reference to calculate the prognostic scores, although other forms of normalization are also possible [19].

Predicted probabilities of the outcome in the development and validation cohorts were calculated by fitting logistic regression models with the prognostic score as the only independent variable in each imputed dataset and using Rubin's rules to combine the results [15]. Discrimination of the prognostic index was assessed using the area under the receiver operating characteristic curve (AUROCC) with confidence intervals (CIs) obtained through 2000 bootstrap samples [5]. Calibration was assessed with the Brier score and graphically by inspecting the smoothed relationship between the predicted and observed risk [5]. Clinical utility was assessed using decision curve analysis [20]. As the concept of net benefit can be difficult to grasp [21], we described a hypothetical scenario where the prognostic index was used to decide whether patients needed admission to the hospital.

Risk groups were formed based on the predicted probability of the outcome: low risk (<5%), intermediate-low risk (5–10%), intermediate-high risk (10–20%), high risk (20–40%), and very high risk (>40%). We performed several sensitivity analyses. We checked the performance of the model using complete case data, segregated by gender, and using mortality as the outcome.

This was an urgent public health research study in response to a Public Health Emergency of International Concern. Patients or the public were not involved in the design, conduct, interpretation, or presentation of the results of this research.

## 3. Results

### 3.1. Model Development

**Figure 1:**
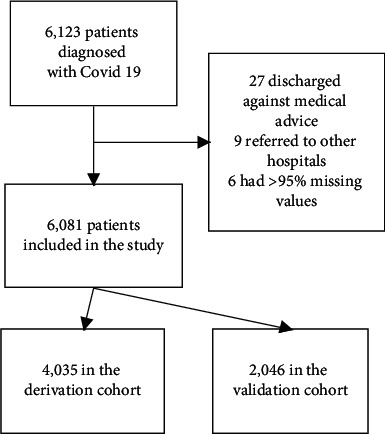
Flowchart of patients.

During the study period, 6123 patients with COVID-19 were admitted to the hospital (Figure 1). Forty-two patients were excluded because they were self-discharged against medical advice (27), were referred to another hospital (9), or had >95% missing values (6). 4035 patients were included in the development cohort and 2046 in the validation cohort. The overall average hospital length of stay was 6.92 days (median 6, interquartile range (IQR) 4 to 8). The median age was 48 years (IQR 34 to 59) and 2348 (38.6%) were female. Differences between the development and the validation cohort are described in Table 1. The primary outcome occurred in 961 (23.8%) patients in the development cohort and in 548 (26.8%) in the validation cohort.

The model development is described in detail in the Supplementary Materials section. From the initial 20 predictor candidates, seven were excluded at the initial stage because of low predictive power or collinearity (Table S1). All remaining predictors were continuous variables and were categorized using GAM to select the optimal cutoff values (Figures S1–S3 and Table S2). The final selection of cutoff values and variables was performed using LASSO logistic regression (Table S3), and coefficients were used to produce the prognostic scores (Table S4). The prognostic index ranged from 0 to 32 and included 12 variables: age, systolic blood pressure, heart rate, respiratory rate, aspartate transaminase, lactate dehydrogenase, urea, C-reactive protein, sodium, absolute lymphocyte count, absolute neutrophil count, and neutrophil/lymphocyte ratio (Table 2). Table 2 also includes the reference range in our laboratory and suggested normalization values based on the upper and lower normal limits.

In the development cohort, the AUROCC was 0.907 (95% CI, 0.896–0.918) (Figure S4), and the Brier score was 0.0935. The calibration-in-the-large was 0.01, and the slope was 1.007 (Figure S5). Based on the predicted probability of the outcome, we created the following risk groups: low risk (index 0, 1, or 2), intermediate-low risk (index 3 or 4), intermediate-high risk (index 5 or 6), high risk (index 7 or 8), and very high risk (index 9 or above) (Table 3).

### 3.2. Model Validation

**Figure 2:**
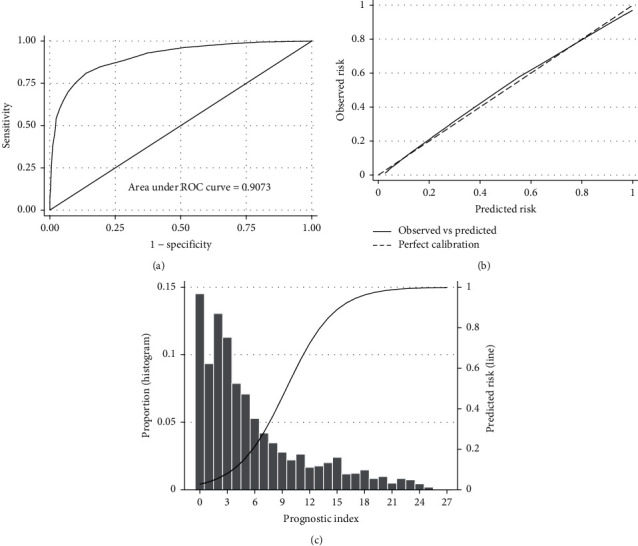
Discrimination (a), calibration (b), distribution of cases (c), and predicted probability of death or hypoxaemia (lower panel line) of the predictive model in the validation cohort.

**Figure 3:**
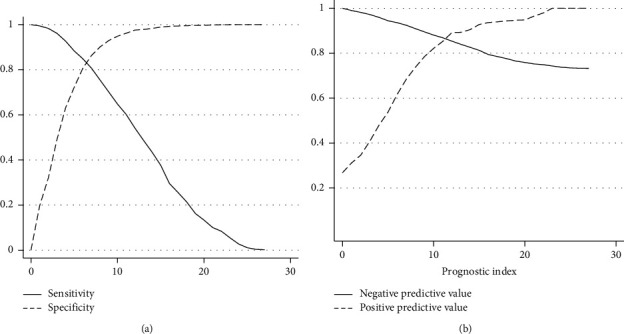
Sensitivity and specificity (a) and negative and positive predictive value (b) in the validation cohort. *Y*-axis represents proportions; *X*-axis represents the prognostic index.

In the validation cohort, the AUROCC was 0.907 (95% CI, 0.892–0.922) and the Brier score was 0.098 (Figure 2(a)). The calibration-in-the-large was 0 (95% CI, −0.14 to 0.14) and the slope was 1 (95% CI, 0.91–1.09) (Figure 2(b)). Nearly 50% of cases had a prognostic index of 3 or less (Figure 2(c) and Table S5). The predicted risk for the primary outcome increased rapidly for prognostic scores between 5 and 10 and then had a progressive reduction (Figure 2(c), Figure S6, and Table S5). Sensitivity, specificity, negative predictive value, and positive predictive value of the prognostic model in the validation cohort are presented in Figure 3. In general, the proportion of patients with outcome by risk group was slightly larger than in the development cohort (Table 3).

**Figure 4:**
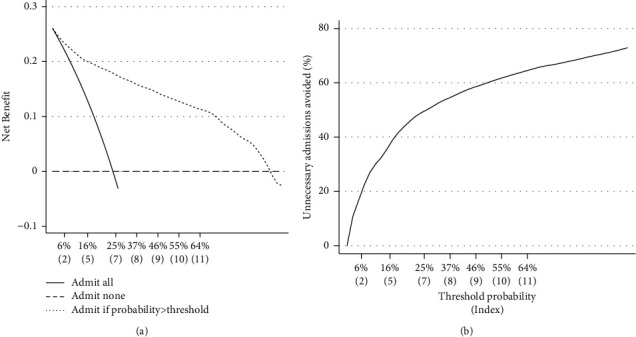
Decision curves. Net benefit (a) and number of interventions avoided (b) in the validation cohort. *Y*-axis represents the threshold probability of hypoxaemia or death given by the prognostic index score, which is mentioned below between brackets.

Decision curve analysis is reported in Figure 4. The net benefit describes the performance of the model to identify true positives over true negatives [21]. Decisions curve analysis can be used to decide whether to initiate an intervention (e.g., to start a particular medication or to request a diagnostic test). However, to better understand the clinical use of the predictive model, we created a hypothetical scenario where patients were admitted (the “intervention”) according to the predicted probability of the outcome given by the prognostic index. We compared the performance of the model with two other possible scenarios: admit all patients (unlimited resources) or admit none (there are no free beds in the hospital). These two scenarios represent extreme situations that can occur in the real world. If the bed occupancy is high because of a sudden spike in the number of COVID-19 cases, we could select a higher prognostic index threshold for admission to optimize resources. If the incidence of COVID-19 cases comes down and the bed occupancy is low, we could be more permissible and reduce the prognostic score cutoff for admission. The selection of the threshold probability represents the trade-off between the benefit and the cost of the intervention. The net benefit of the predictive model was positive and above the net benefit of other alternatives (admit all or admit none) up to threshold probabilities above 90%, which are hardly justifiable in real life (Figure 4(a)). If we consider patients who did not develop the outcome and did not need admission, the number of unnecessary admissions avoided is presented in Figure 4(b). For example, by using a prognostic index of 7 or more as the threshold for admission, we could have reduced nearly 50% of the number of admissions.

**Figure 5:**
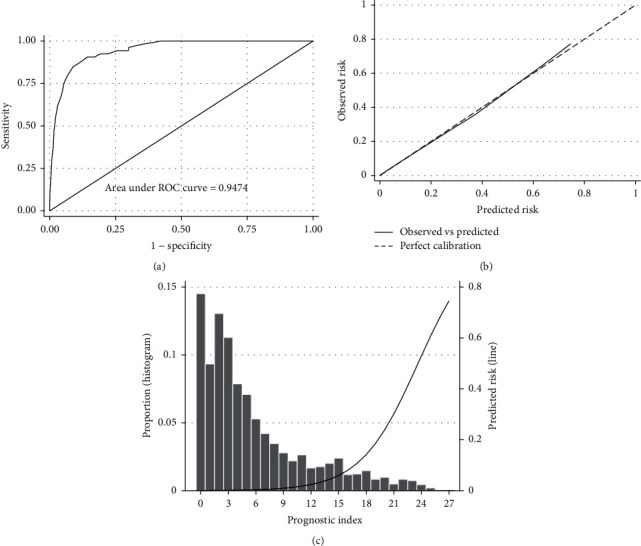
Discrimination (a), calibration (b), distribution of cases (c), and predicted probability (lower panel line) of the model to predict mortality in the validation cohort.

In a sensitivity analysis using only complete cases, the results were almost identical (AUROCC was 0.907, 95% CI, 0.893–0.922; Brier score 0.0977). The model performed slightly better in female cases (AUROCC 0.921, 95% CI, 0.896–0.945; Brier score 0.078) than in male cases (AUROCC 0.899, 95% CI, 0.88–0.918; Brier score 0.109). The prognostic index showed excellent accuracy (AUROCC 0.947; 95% CI, 0.925–0.97) and calibration (Brier score 0.0188; calibration-in-the-large 0.0006, 95% CI, −0.315 to 0.316; slope 1.001, 95% CI, 0.816–1.188) to predict mortality. The performance of the model to predict mortality is described graphically in Figure 5 and Figures S7-S8.

## 4. Discussion

In this study, we present a pragmatic multivariable prognostic index that showed good discrimination calibration and clinical utility in a cohort that could be considered representative of COVID-19 cases diagnosed in the community. The variables included in the RCOS prognostic index are readily available in most healthcare settings with basic laboratory infrastructure, and the index does not require complex calculations. It could be used to optimize resources in overwhelmed health systems, identifying patients who are more likely to develop complications and need hospital admission or closer ambulatory monitoring. Optimal utilization of resources is especially important in low- and middle-income countries, where public health facilities are overburdened and unable to accommodate the high number of cases who need hospitalization in the current COVID-19 pandemic.

Current therapy of COVID-19 focuses on patients who have already developed complications [22]. Previous studies have shown that the highest level of viral replication occurs around the first day of symptoms, and 95% of hospitalized patients have negative viral cultures after 15 days of symptoms [23]. Current evidence suggests that antiviral and antibody therapy are more effective if started early, during the first days of symptoms [24, 25]. The RCOS prognostic index could be used to escalate therapy in patients with a higher risk of complications. The index could also help identify high-risk groups in targeted randomized clinical trials investigating early interventions aimed to reduce morbidity or mortality of COVID-19.

### 4.1. Strengths and Limitations

In COVID-19 cases, hypoxaemia usually appears within five to ten days of symptoms [26–28]. In our study, patients were admitted regardless of the severity of symptoms and were not discharged before ten days passed from symptom onset. In the development cohort, 23.8% of the patients developed hypoxaemia or died, which is similar to the proportion of severe cases found in a large cohort from China [2]. This suggests that the model was developed in a population representative of COVID-19 in the community. However, the validation cohort had a larger proportion of patients with hypoxaemia than the development cohort, and both predicted and observed risks were higher than expected. This can be explained by the fact that the COVID-19 incidence increased during the study period, leading to increased hospital pressure from more severe cases. When implementing the prognostic index in populations with lower (e.g., primary health center) or higher (e.g., emergency department) expected risk, the use of risk groups may overestimate (primary health center) or underestimate (emergency department) the real risk of complications. Although classifying patients in risk groups can still be useful as an initial reference, our results suggest that users of the prognostic index should try to estimate the predicted probability of the outcome in their settings, especially in populations with high rates of SARS-CoV-2 vaccination.

The prognostic model was not developed to predict complications in hospital settings with high mortality. Still, the excellent performance of the prognostic index to predict mortality suggests that it could be a helpful companion to other severity predictors such as oxygen saturation or PaO_2_/FiO_2_ ratio to identify patients who are more likely to require a ventilator or critical care support [29], but new studies are needed to confirm this hypothesis.

The study has several limitations. Unlike other COVID-19 prognostic models, the RCOS prognostic index does not include comorbid conditions of the patients [4]. It is possible that including comorbidity predictors could improve the performance of the model. This is a single-centre study, and validation was performed in the same setting as the development. However, we used data from different periods of time to validate our model, which is a stronger approach compared to other forms of internal validation, and can be considered intermediate between internal and external validation [5]. In addition, the score relies on laboratory data that might be difficult to perform in primary health centres.

## 5. Conclusion

Prognostic models are able to transform complex clinical situations into a single dimension numerical value. In this study, we present a prognostic score that demonstrated excellent discrimination and calibration to predict complications and mortality in a population of COVID-19 cases that included a large proportion of mild cases. If our results are validated in other settings, the RCOS prognostic index could help optimize resources in overstretched healthcare systems and improve clinical decisions in COVID-19 patients diagnosed in the community who are at higher risk of developing complications. A preprint has previously been published [30]

## Figures and Tables

**Table 1:** Characteristics of patients in the development and the validation cohorts.

	Development cohort		Validation cohort	
	Median (IQR) or no (%)	Missing	Median (IQR) or no (%)	Missing
Age (years)	48 (34–60)	9	47 (33–58)	0
Systolic BP (mm Hg)	120 (110–120)	1111	120 (110–120)	4
Diastolic BP (mm Hg)	80 (70–80)	1111	80 (70–80)	4
Heart rate (min)	88 (82–92)	1112	86 (80–90)	5
Respiratory rate (min)	20 (20–22)	1117	20 (20–22)	5
Temperature (°F)	99 (99–99)	1110	99 (98–99)	5
AST (IU/L)	25 (18–36)	16	22 (17–31)	3
ALT (IU/L)	27 (18–42)	18	26 (18–40)	3
Albumin (g/dL)	4.5 (4.2–4.8)	18	4.6 (4.3–4.9)	3
LDH (IU/L)	385 (269–495)	7	375 (284–467)	1
Creatinine (mg/dL)	0.8 (0.7–1)	6	0.7 (0.6–0.8)	3
Urea (mg/dL)	22 (17–29)	7	22 (18–29)	3
C-reactive protein (mg/dL)	0.5 (0.2–3)	5	0.5 (0.3–2.8)	1
Sodium (mmol/L)	141 (139–143)	5	141 (139–143)	3
Haemoglobin (g/dL)	13 (12–14)	82	13 (12–14)	0
Platelet count (×10^9/L)	265 (207–333)	82	304 (243–364)	0
White cell count (×10^9/L)	7 (5.5–9.1)	82	6.9 (5.4–9)	0
Neutrophil count (×10^9/L)	4.5 (3.3–6.2)	82	4.4 (3.3–6.2)	0
Lymphocyte count (×10^9/L)	1.9 (1.4–2.6)	82	1.9 (1.4–2.4)	0
Neutrophil/Lymphocyte ratio	2.3 (1.6–3.7)	82	2.3 (1.6–3.8)	0
Female gender	1593 (39.5)	0	752 (36.8)	0
Deaths	172 (4.3)	0	53 (2.6)	0
Hypoxaemia	959 (23.8)	0	545 (26.6)	0

IQR, interquartile range; BP, blood pressure; ALT, alanine transaminase; AST, aspartate transaminase; LDH, lactate dehydrogenase.

**Table 2:** The RCOS prognostic index.

	Reference range	Prognostic score
Age (years)		
40–49		1
50–59		2
60–69		3
≥70		4
Systolic BP (mm Hg) ≥ 140		1
Heart rate (pm) ≥ 100		1
Respiratory rate (pm) ≥ 22		2
AST (IU/L)	0 to 40	
40–79	1 to 2x UNL	1
≥80	>2x UNL	2
LDH (IU/L)	207 to 414	
700–899	1.69 to 2.17x UNL	1
≥900	>2.17x UNL	2
Urea (mg/dL)	15 to 39	
40–49.9	1 to 1.25x UNL	2
≥50	>1.25x UNL	3
C-reactive protein (mg/dL)	0 to 0.5	
0.5–0.9	1 to 1.99x UNL	1
1–1.9	2 to 3.99x UNL	2
2–3.9	4 to 7.99x UNL	3
4–5.9	8 to 11.99x UNL	4
6–8.9	12 to 17.99x UNL	5
9–11.9	18 to 23.99x UNL	6
≥12	≥24x UNL	7
Sodium-mmol/L < 135	135 to 148	1
Lymphocyte count (×10^9/L)	1 to 5	
<0.8		3
0.8–0.999		1
Neutrophil count (×10^9/L)	1.2 to 8	
8–9.9		1
≥10		2
Neutrophil/lymphocyte ratio		
3–3.9		1
4–5.9		2
6–7.9		3
≥8		4

BP, blood pressure; pm, per minute; AST, aspartate transaminase; LDH, lactate dehydrogenase; UNL, upper normal limit; LNL lower normal limit.

**Table 3:** Proportion of patients who experienced the study outcome (death or hypoxaemia) segregated by risk group.

Risk group	Development		Validation	
	No. of patients (%)	Outcome (%)	No of. patients (%)	Outcome (%)
Low (0–2)	1314 (32.57)	37 (2.82)	755 (36.9)	21 (2.78)
Intermediate-low (3–4)	832 (20.62)	36 (4.33)	392 (19.16)	43 (10.97)
Intermediate-high (5–6)	533 (13.21)	70 (13.13)	253 (12.37)	42 (16.6)
High (7–8)	340 (8.43)	95 (27.94)	157 (7.67)	58 (36.94)
Very high (>8)	1016 (25.18)	723 (71.16)	489 (23.9)	384 (78.53)

## Data Availability

The conditions of the ethical approval for the study preclude open access to data sharing to reduce the risk of patient identification. Specific requests for data sharing will be considered subject to ethical approval and data transfer agreements.

## Supplementary Materials

Supplementary Materials Additional information about the methods of the study and TRIPOD statement.
