# Supplementary material for: Development and Validation of the RCOS Prognostic Index: A Bedside Multivariable Logistic Regression Model to Predict Hypoxaemia or Death in Patients with SARS-CoV-2 Infection
Source: Interdiscip Perspect Infect Dis. 2022 Apr 20;2022:2360478. doi: 10.1155/2022/2360478 (PMC9020413; doi:10.1155/2022/2360478)
Supplement: Supplementary Materials — Additional information about the methods of the study and TRIPOD statement. [file 2360478.f1.pdf]

Supplementary material

Contents

Software 2

Model development 2

Stage 1 2

Stage 2 3

Stage 3 6

Stage 4 7

Model performance in the development cohort 8

Predicted risk given by prognostic scores in the validation cohort 9

Model performance to predict mortality in the validation cohort 11

References 13

TRIPOD checklist 14

Software

Statistical analysis was performed using Stata 14.2, except for generalised additive models (GAM),which were performed in R (version 4.0.3) with the mgcv library (version 1.8-33) [1]. Missing data were handled by multiple imputation using chained equations and predictive mean matching (with five nearest neighbours) for continuous variables in 10 datasets, each with 10 iterations [2, 3]. The selection of variables for the final model was performed using least absolute shrinkage and selection operator (LASSO) logistic regression with theory-driven penalization, which have shown to reduce the risk of overfitting compared with other penalization methods (rlassologit command from the lassopack version 1.4.1) [4]. Calibration slopes were calculated with the coefficient of a logistic model for the outcome and the model linear predictor as the independent variable; and calibration-in-the-large was calculated with the intercept of a logistic model for the outcome with the model linear predictor as an offset term.

Model development

Our aim was to construct a simple predictive score that could be used bedside by clinicians without the need of computers or mobile applications. The model was developed in four stages.

Stage 1

We assessed the goodness of fit between the outcome and predictors using GAM models. We excluded predictors with a deviance explained below 2% (female gender, diastolic blood pressure, temperature, haemoglobin concentration and platelet count). To avoid multicollinearity problems, when two predictors were highly correlated (such as Alanine transaminase (ALT) and Aspartate transaminase (AST); and white cell count and neutrophil count), we excluded the ones that had lower goodness of fit (ALT and white cell count).

Table S1.

Predictors	R^2	DE	REML	
Female gender	0.011	1.1%	2195.1	Excluded
Age-years	0.107	10.7%	1979.4	
Systolic BP-mm Hg	0.050	4.0%	1754.8	
Diastolic BP-mm Hg	0.009	0.8%	1810.2	Excluded
Heart rate-min	0.030	2.4%	1781.4	
Respiratory rate-min	0.103	8.0%	1675.4	
Temperature-°F	0.017	1.6%	1802.0	Excluded
AST-IU/L	0.084	7.3%	2057.6	
ALT-IU/L	0.041	3.7%	2136.7	Excluded
Albumin-g/dL	0.144	13.1%	1925.6	
LDH- IU/L	0.147	12.1%	1959.7	
Creatinine-mg/dL	0.076	6.8%	2072.6	
Urea-mg/dL	0.169	13.8%	1919.9	
C-reactive protein-mg/dL	0.370	32.0%	1518.9	
Sodium-mmol/l	0.156	13.4%	1933.0	
Haemoglobin-g/dL	0.002	0.3%	2185.6	Excluded
Platelet count-×10^9/L	0.010	0.9%	2174.8	Excluded
White cell count-×10^9/L	0.081	6.4%	2056.5	Excluded
Neutrophil count-×10^9/L	0.129	10.3%	1970.5	
Lymphocyte count-×10^9/L	0.165	14.2%	1886.9	
Neutrophil/Lymphocyte ratio	0.272	23.1%	1691.2	

DE, deviance explained, REML, restricted maximum likelihood; BP, blood pressure; ALT, Alanine transaminase; AST, Aspartate transaminase; LDH, Lactate dehydrogenase.

Stage 2

We selected optimal cut-off values to categorize continuous variables based on visual inspection of the GAM models [5], taking into account clinically important points, the laboratory reference range for normal values (we avoided cut-off values within the normal range), cut-off values used in other risk scores, and the distribution of values in the dataset (we avoided placing cut-off values far below the percentile 5 or far above the percentile 95). To minimise the loss of information produced by categorizing continuous variables, we tried to keep similar "risk-distance" between cut-off values (Figures S1-S3 and Table S2).

Figure S1. GAM models 1. DE=deviance explained. Vertical lines represent percentile 50 (solid line); 25 and 75 (dashed line ----); 10 and 90 (dotted line …..); 5 and 95 (dashed/dotted lines _._._). Risk denotes the log-odds of the outcome (hypoxaemia or death).

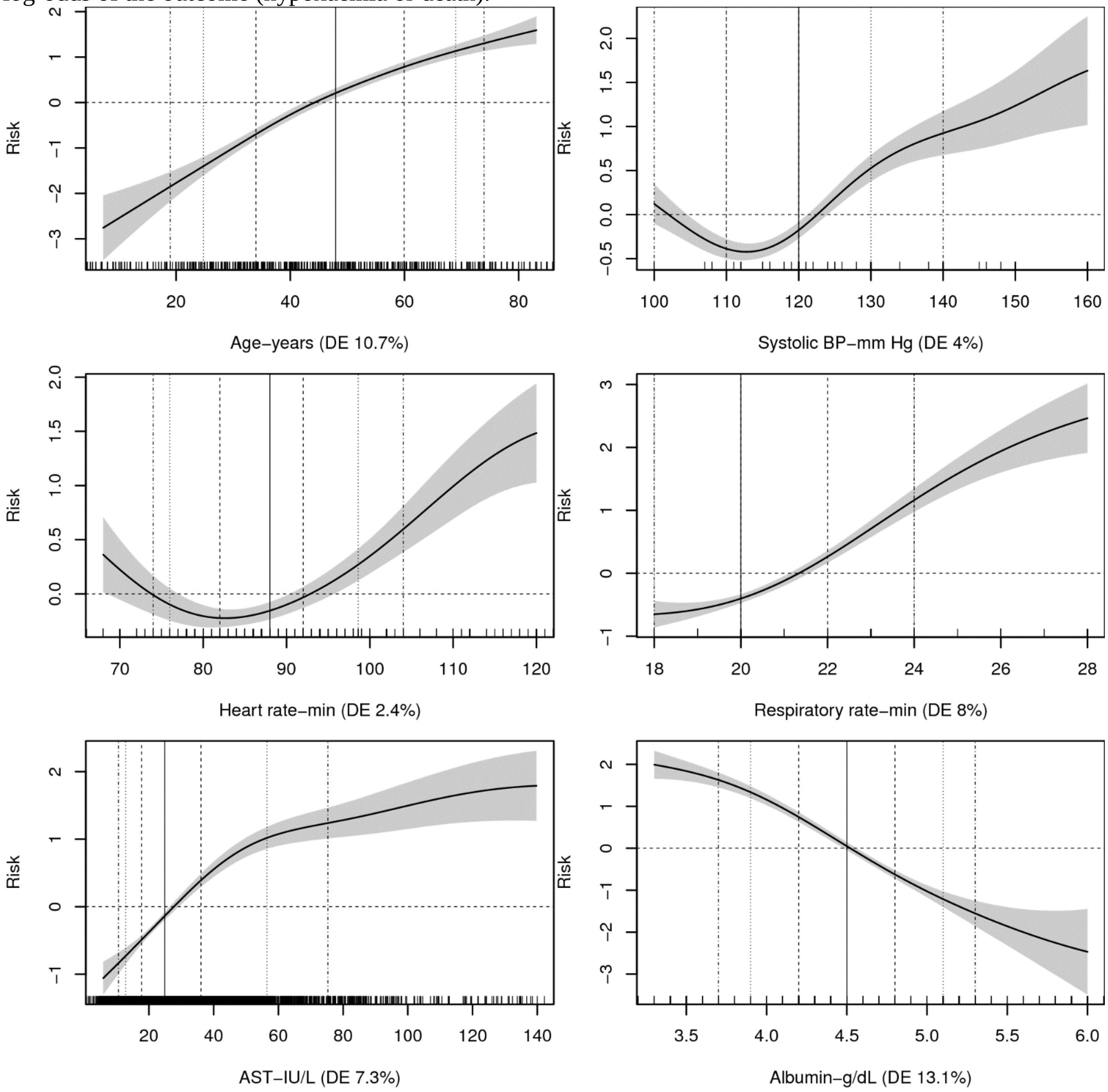

Figure S2. GAM models 2. DE=deviance explained. Vertical lines represent percentile 50 (solid line); 25 and 75 (dashed line ----); 10 and 90 (dotted line …..); 5 and 95 (dashed/dotted lines _._._)

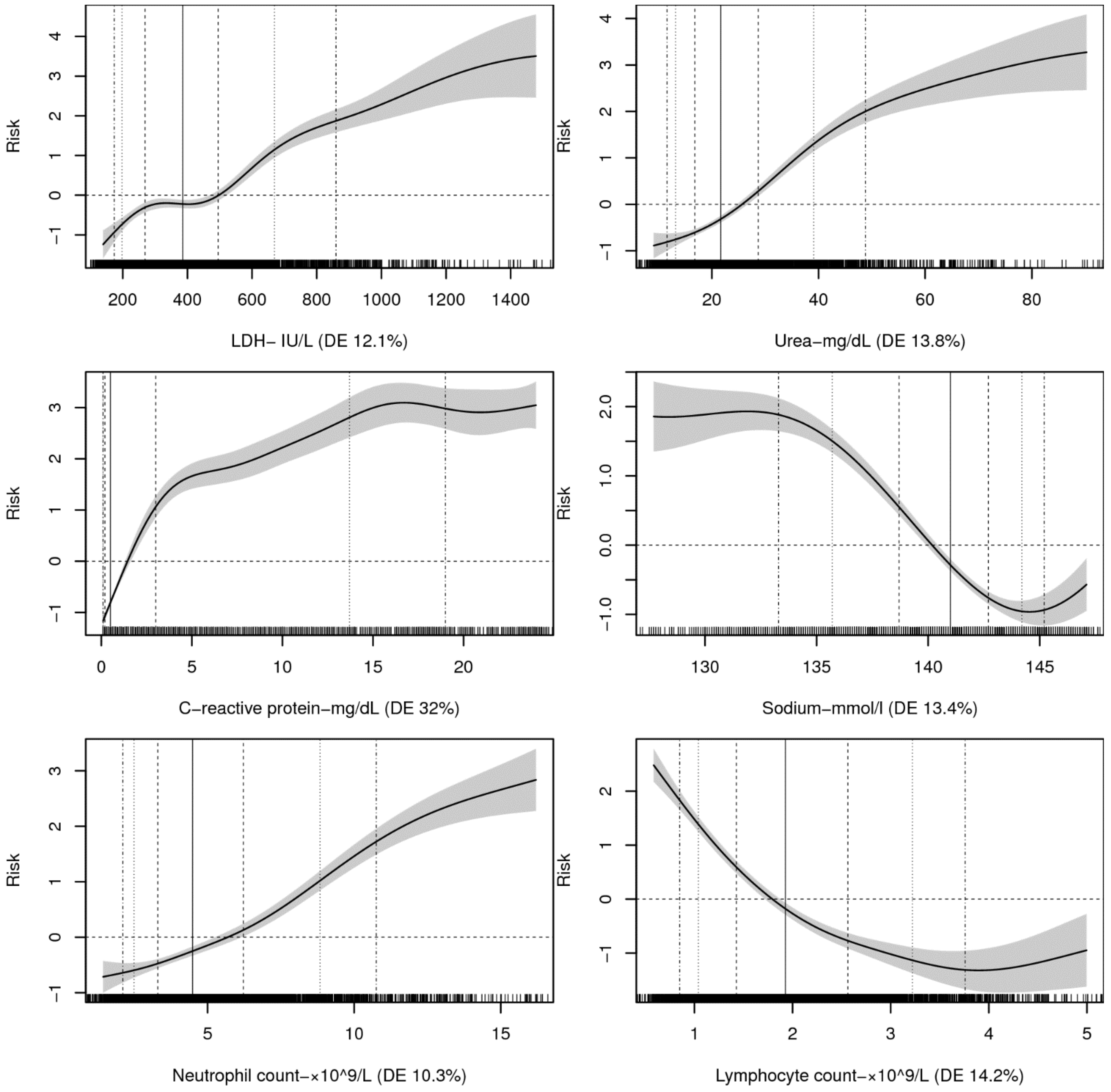

Figure S3. GAM models 3. DE=deviance explained. Vertical lines represent percentile 50 (solid line); 25 and 75 (dashed line ----); 10 and 90 (dotted line …..); 5 and 95 (dashed/dotted lines _._._)

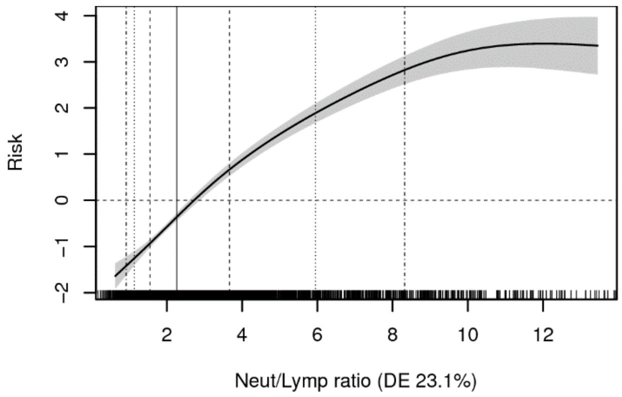

Table S2. Selected cut-off values for continuous variables.

	Laboratory NR	Cut-off
Age-years	NA	40,50,60,70
Systolic BP-mm Hg	NA	140
Heart rate-min	NA	100
Respiratory rate-min	NA	22
AST-IU/L	0-40	40, 80
Albumin-g/dL	3.5-5.3	3.5
LDH- IU/L	207-414	400, 700, 900
Urea-mg/dL	15-39	40, 50
C-reactive protein-mg/dL	0-0.5	0.5,1,2,4,6,9,12
Sodium-mmol/L	135-148	135
Neutrophil count-×10^9/L	1.2-8	8, 10
Lymphocyte count-×10^9/L	1-5	0.8, 1
Neutrophil/Lymphocyte ratio	NA	3,4,6,8

NR, normal range; BP, blood pressure; AST, Aspartate transaminase; LDH, Lactate dehydrogenase

Stage 3

Using the initial cut-off values selected in Stage 2, we performed LASSO logistic regression for each imputed dataset looking for overall agreement to select the cut-off values for the final model [2]. There was a 100% agreement among the imputed datasets. All initial cut-off values were included by the LASSO model except albumin (which was excluded from the final model) and LDH 400 IU/L. See table S3.

Table S3. Selection of cut-off values using LASSO regression.

	Initial selection (Stage 2)	LASSO selection
Age-years	40,50,60,70	40,50,60,70
Systolic BP-mm Hg	140	140
Heart rate-min	100	100
Respiratory rate-min	22	22
AST-IU/L	40, 80	40, 80
Albumin-g/dL	3.5	Excluded
LDH- IU/L	400, 700, 900	700, 900
Urea-mg/dL	40, 50	40, 50
C-reactive protein-mg/dL	0.5,1,2,4,6,9,12	0.5,1,2,4,6,9,12
Sodium-mmol/L	135	135
Neutrophil count-×10^9/L	8, 10	8, 10
Lymphocyte count-×10^9/L	0.8, 1	0.8, 1
Neutrophil/Lymphocyte ratio	3,4,6,8	3,4,6,8

BP, blood pressure; AST, Aspartate transaminase; LDH, Lactate dehydrogenase

Stage 4

We combined logistic regression models from the imputed datasets using Rubin's rules. Coefficients from this logistic model and LASSO penalised coefficients were combined and scaled (x3) to produce the prognostic index.

Table S4. LASSO regression, logistic regression coefficients and final prognostic index.

	Penalised coefficient	Logit coefficient (95% CI)	Prognostic score
Age (years)			
40-49	0.16	0.65 (0.32 to 0.98)	1
50-59	0.53	0.98 (0.67 to 1.29)	2
60-69	0.76	1.24 (0.91 to 1.58)	3
>=70	0.87	1.38 (0.97 to 1.8)	4
Systolic BP (mm Hg)			
>= 140	0.32	0.39 (0.02 to 0.76)	1
Heart rate (pm)			
>=100	0.19	0.31 (-0.04 to 0.65)	1
Respiratory rate (pm)			
>=22	0.64	0.77 (0.54 to 1)	2
AST-IU/L			
40-79	0.38	0.43 (0.17 to 0.69)	1
>=80	0.61	0.85 (0.38 to 1.32)	2
LDH- IU/L			
700-899	0.3	0.37 (-0.05 to 0.8)	1
>=900	0.58	0.71 (0.2 to 1.23)	2
Urea-mg/dL			
40-49.9	0.54	0.61 (0.21 to 1.01)	2
>=50	0.95	1.01 (0.55 to 1.48)	3
C-reactive protein-mg/dL			
0.5-0.9	0.23	0.73 (0.37 to 1.09)	1
1-1.9	0.61	1.04 (0.69 to 1.4)	2
2-3.9	0.93	1.32 (0.96 to 1.68)	3
4-5.9	1.44	1.83 (1.4 to 2.25)	4
6-8.9	1.82	2.24 (1.79 to 2.69)	5
9-11.9	2.05	2.47 (1.99 to 2.95)	6
>=12	2.46	2.8 (2.44 to 3.16)	7
Sodium-mmol/L			
<135	0.49	0.46 (0.14 to 0.79)	1
Lymphocyte count-×10^9/L			
<0.8	0.86	1.06 (0.54 to 1.58)	3
0.8-0.99	0.24	0.32 (-0.11 to 0.75)	1
Neutrophil count-×10^9/L			
8 - 9.9	0.1	0.25 (-0.17 to 0.66)	1
>=10	0.67	0.88 (0.37 to 1.39)	2
Neutrophil/Lymphocyte ratio			
3-3.9	0.25	0.36 (0.07 to 0.65)	1
4-5.9	0.47	0.52 (0.19 to 0.84)	2
6-7.9	0.77	0.83 (0.32 to 1.35)	3
>=8	1.07	1.15 (0.52 to 1.77)	4

Model performance in the development cohort

Figure S4. Discrimination in the development cohort.

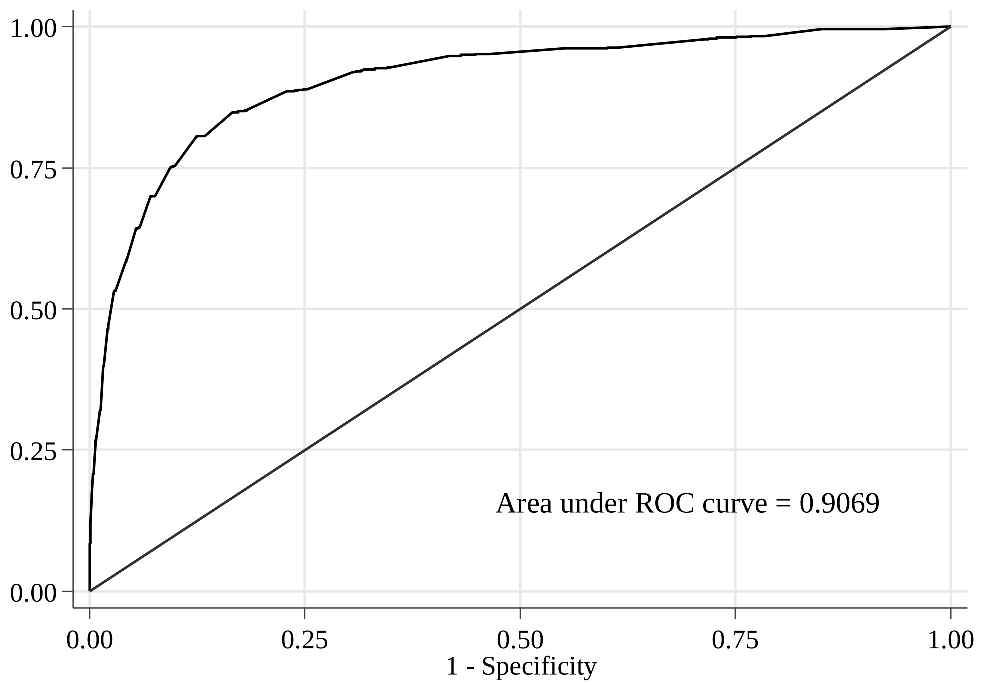

Figure S5. Calibration in the development cohort.

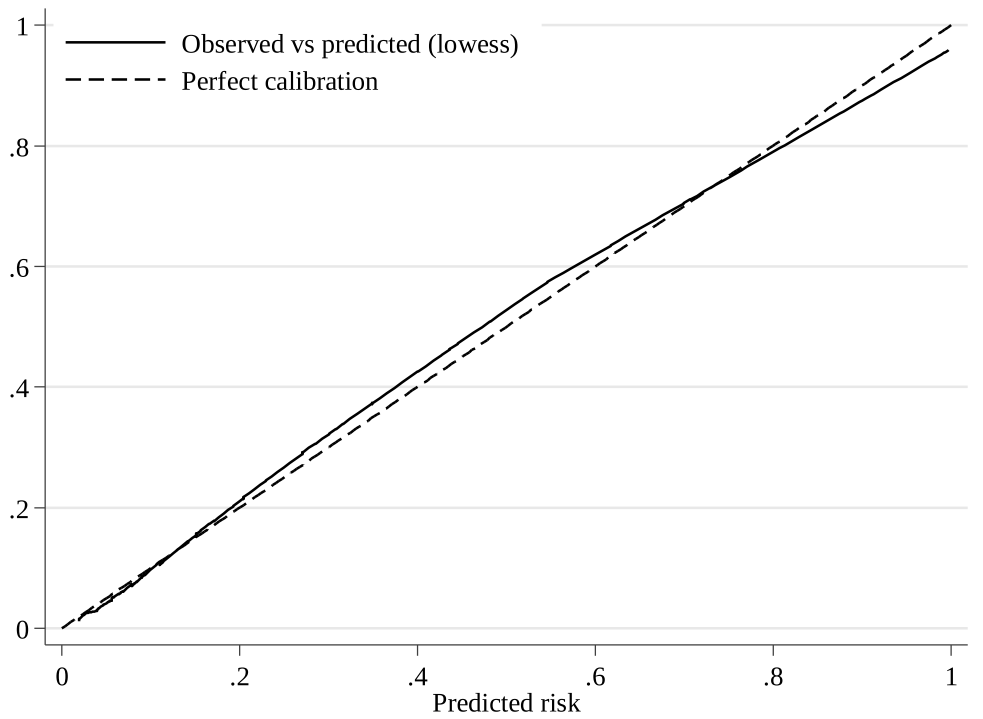

Predicted risk given by prognostic scores in the validation cohort

Table S5. Distribution of patients and predicted risk of the outcome (hypoxaemia or death) and mortality in the validation cohort.

	Distribution			Predicted risk	
Prognostic index	N	Percent	Cumulative percent	Outcome	Mortality
0	297	14.52	14.52	2.74%	0.05%
1	191	9.34	23.85	3.95%	0.07%
2	267	13.05	36.9	5.66%	0.10%
3	231	11.29	48.19	8.05%	0.14%
4	161	7.87	56.06	11.31%	0.19%
5	145	7.09	63.15	15.68%	0.26%
6	108	5.28	68.43	21.35%	0.35%
7	86	4.2	72.63	28.34%	0.49%
8	71	3.47	76.1	36.58%	0.67%
9	57	2.79	78.89	45.68%	0.92%
10	45	2.2	81.09	55.09%	1.26%
11	54	2.64	83.72	64.14%	1.73%
12	34	1.66	85.39	72.28%	2.36%
13	36	1.76	87.15	79.18%	3.23%
14	41	2	89.15	84.72%	4.39%
15	49	2.39	91.54	88.99%	5.94%
16	24	1.17	92.72	92.18%	8.00%
17	25	1.22	93.94	94.50%	10.70%
18	30	1.47	95.41	96.16%	14.16%
19	17	0.83	96.24	97.34%	18.51%
20	20	0.98	97.21	98.16%	23.83%
21	10	0.49	97.7	98.73%	30.11%
22	17	0.83	98.53	99.13%	37.23%
23	15	0.73	99.27	99.40%	44.95%
24	9	0.44	99.71	99.59%	52.93%
25	4	0.2	99.9	99.72%	60.76%
26	1	0.05	99.95	99.81%	68.07%
27	1	0.05	100	99.87%	74.59%

Figure S6. Percentage risk increase by one point increase in the prognostic index.

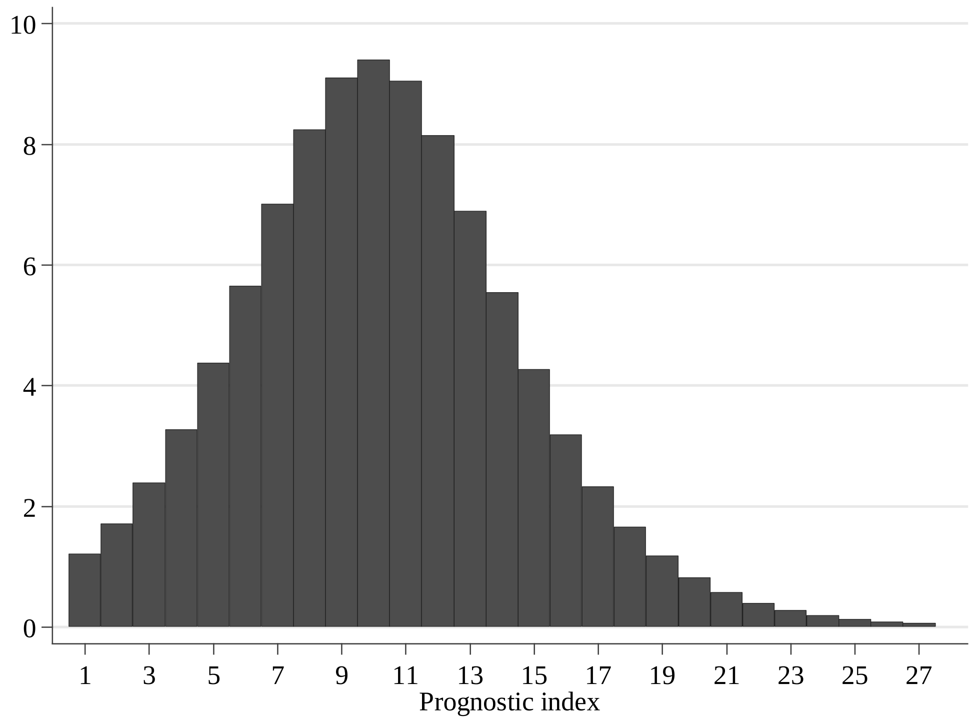

Model performance to predict mortality in the validation cohort

Figure S7. Sensitivity, specificity, negative predictive value and positive predictive value of the predictive model to predict mortality in the validation cohort.

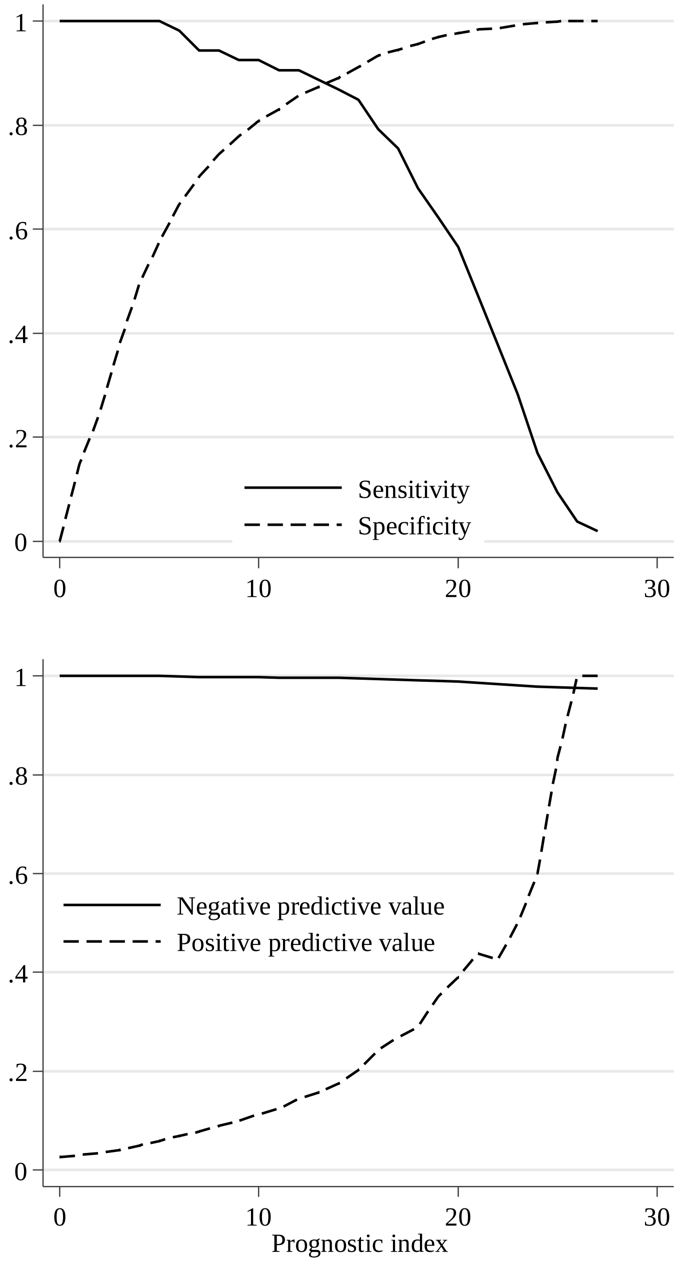

Figure S8. Decision curves. Net benefit (upper panel) and number of intervention avoided (lower panel) of the prognostic model for mortality in the validation cohort.

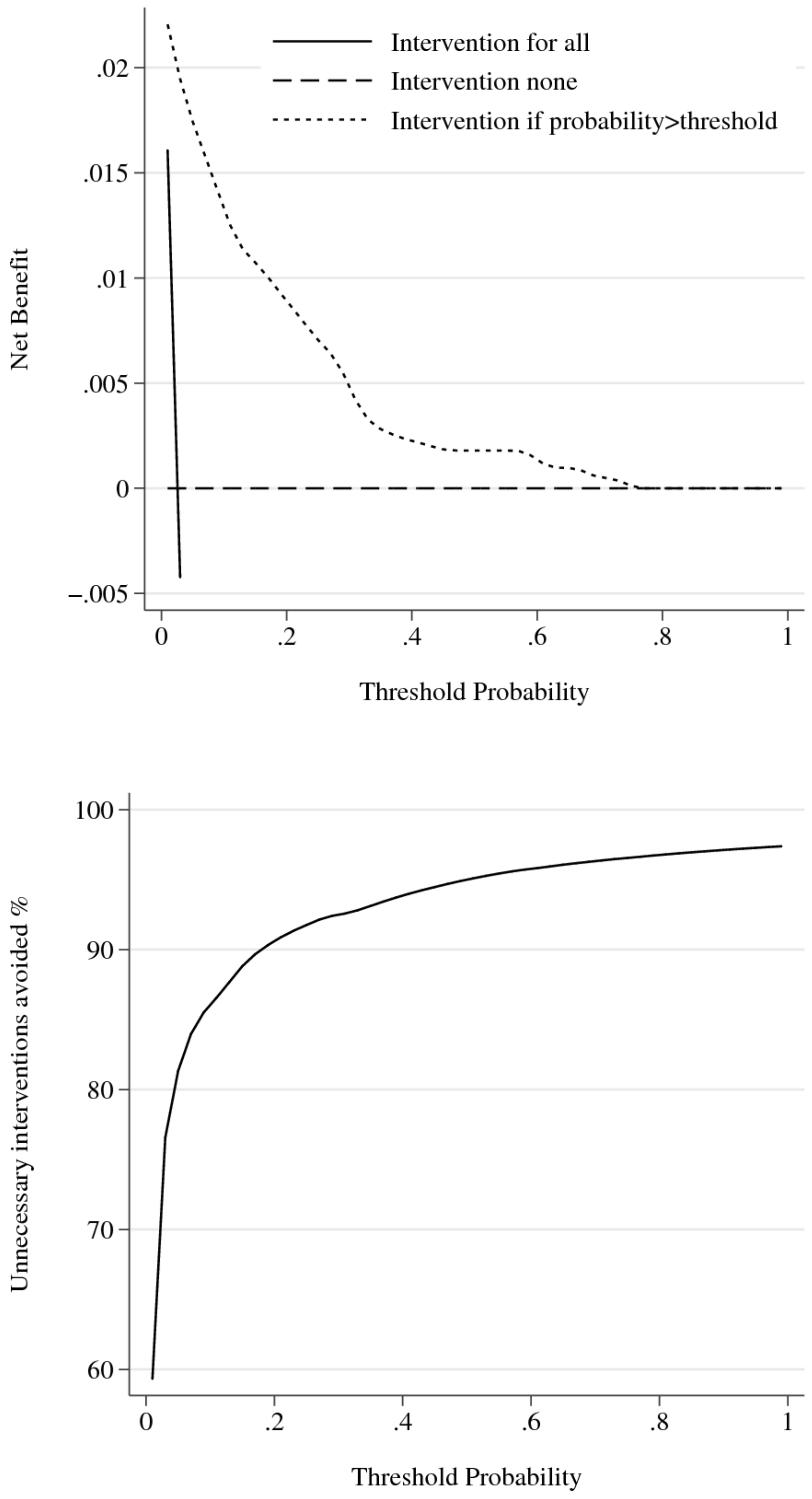

References

1. Wood SN. Generalized additive models: an introduction with R. CRC press; 2017.

2. Wood AM, White IR, Royston P. How should variable selection be performed with multiply imputed data? Stat Med. 2008;27:3227–46.

3. Morris TP, White IR, Royston P. Tuning multiple imputation by predictive mean matching and local residual draws. BMC Med Res Methodol. 2014;14:75.

4. Ahrens A, Hansen CB, Schaffer ME. lassopack: Model selection and prediction with regularized regression in Stata. Stata J. 2020;20:176–235.

5. Barrio I, Arostegui I, Quintana JM, Group I-C. Use of generalised additive models to categorise continuous variables in clinical prediction. BMC Med Res Methodol. 2013;13:83.

TRIPOD checklist

Section/Topic			Checklist Item	Page
**Title and abstract**				
Title	1	D;V	Identify the study as developing and/or validating a multivariable prediction model, the target population, and the outcome to be predicted.	1
Abstract	2	D;V	Provide a summary of objectives, study design, setting, participants, sample size, predictors, outcome, statistical analysis, results, and conclusions.	2
**Introduction**				
Background and objectives	3a	D;V	Explain the medical context (including whether diagnostic or prognostic) and rationale for developing or validating the multivariable prediction model, including references to existing models.	3
	3b	D;V	Specify the objectives, including whether the study describes the development or validation of the model or both.	3
**Methods**				
Source of data	4a	D;V	Describe the study design or source of data (e.g., randomized trial, cohort, or registry data), separately for the development and validation data sets, if applicable.	3-4
	4b	D;V	Specify the key study dates, including start of accrual; end of accrual; and, if applicable, end of follow-up.	3-4
Participants	5a	D;V	Specify key elements of the study setting (e.g., primary care, secondary care, general population) including number and location of centres.	3-4
	5b	D;V	Describe eligibility criteria for participants.	3-4
	5c	D;V	Give details of treatments received, if relevant.	NA
Outcome	6a	D;V	Clearly define the outcome that is predicted by the prediction model, including how and when assessed.	4
	6b	D;V	Report any actions to blind assessment of the outcome to be predicted.	NA
Predictors	7a	D;V	Clearly define all predictors used in developing or validating the multivariable prediction model, including how and when they were measured.	4 & SM
	7b	D;V	Report any actions to blind assessment of predictors for the outcome and other predictors.	5
Sample size	8	D;V	Explain how the study size was arrived at.	4
Missing data	9	D;V	Describe how missing data were handled (e.g., complete-case analysis, single imputation, multiple imputation) with details of any imputation method.	5
Statistical analysis methods	10a	D	Describe how predictors were handled in the analyses.	5&SM
	10b	D	Specify type of model, all model-building procedures (including any predictor selection), and method for internal validation.	5&SM
	10c	V	For validation, describe how the predictions were calculated.	4
	10d	D;V	Specify all measures used to assess model performance and, if relevant, to compare multiple models.	5-6
	10e	V	Describe any model updating (e.g., recalibration) arising from the validation, if done.	NA
Risk groups	11	D;V	Provide details on how risk groups were created, if done.	4
Development vs. validation	12	V	For validation, identify any differences from the development data in setting, eligibility criteria, outcome, and predictors.	6
**Results**				
Participants	13a	D;V	Describe the flow of participants through the study, including the number of participants with and without the outcome and, if applicable, a summary of the follow-up time. A diagram may be helpful.	6& Fig1
	13b	D;V	Describe the characteristics of the participants (basic demographics, clinical features, available predictors), including the number of participants with missing data for predictors and outcome.	Tab1
	13c	V	For validation, show a comparison with the development data of the distribution of important variables (demographics, predictors and outcome).	Tab1
Model development	14a	D	Specify the number of participants and outcome events in each analysis.	6
	14b	D	If done, report the unadjusted association between each candidate predictor and outcome.	SM
Model specification	15a	D	Present the full prediction model to allow predictions for individuals (i.e., all regression coefficients, and model intercept or baseline survival at a given time point).	SM
	15b	D	Explain how to the use the prediction model.	Tab2
Model performance	16	D;V	Report performance measures (with CIs) for the prediction model.	7-9
Model-updating	17	V	If done, report the results from any model updating (i.e., model specification, model performance).	NA
**Discussion**				
Limitations	18	D;V	Discuss any limitations of the study (such as nonrepresentative sample, few events per predictor, missing data).	9-10
Interpretation	19a	V	For validation, discuss the results with reference to performance in the development data, and any other validation data.	7-9
	19b	D;V	Give an overall interpretation of the results, considering objectives, limitations, results from similar studies, and other relevant evidence.	9-10
Implications	20	D;V	Discuss the potential clinical use of the model and implications for future research.	9
**Other information**				
Supplementary information	21	D;V	Provide information about the availability of supplementary resources, such as study protocol, Web calculator, and data sets.	11
Funding	22	D;V	Give the source of funding and the role of the funders for the present study.	11

*Items relevant only to the development of a prediction model are denoted by D, items relating solely to a validation of a prediction model are denoted by V, and items relating to both are denoted D;V.
